# Succession of the Resident Soil Microbial Community in Response to Periodic Inoculations

**DOI:** 10.1128/AEM.00046-21

**Published:** 2021-04-13

**Authors:** Zhikang Wang, Ziyun Chen, George A. Kowalchuk, Ziheng Xu, Xiangxiang Fu, Eiko E. Kuramae

**Affiliations:** a Co-Innovation Center for Sustainable Forestry in Southern China, College of Forestry, Nanjing Forestry University, Nanjing, China; b Department of Microbial Ecology, Netherlands Institute of Ecology (NIOO-KNAW), Wageningen, the Netherlands; c Ecology and Biodiversity, Institute of Environmental Biology, Utrecht University, Utrecht, the Netherlands; d State Key Laboratory of Soil and Sustainable Agriculture, Institute of Soil Science, Chinese Academy of Sciences, Nanjing, China; University of Michigan—Ann Arbor

**Keywords:** beneficial microorganisms, periodic inoculation, microbial community succession, resident microbiome, inoculant type, soil remediation

## Abstract

To maintain the beneficial effects of microbial inoculants on plants and soil, repeated inoculation represents a promising option. Until now, the impacts of one-off inoculation on the native microbiome have been explored, but it remains unclear how long and to what extent the periodic inoculations would affect the succession of the resident microbiome in bulk soil. Here, we examined the dynamic responses of plant growth, soil functions, and the resident bacterial community in the bulk soil to periodic inoculations of phosphate-solubilizing and N_2_-fixing bacteria alone or in combination. Compared to single-strain inoculation, coinoculation better stimulated plant growth and soil nutrients. However, the benefits from inoculants did not increase with repeated inoculations and were not maintained after transplantation to a different site. In response to microbial inoculants, three patterns of shifts in the bacterial composition were observed: fold increase, fold decrease, and resilience. The periodic inoculations impacted the succession course of resident bacterial communities in bulk soil, mainly driven by changes in soil pH and nitrate, resulting in the development of three main cluster types throughout the investigation. The single and mixed inoculants transiently modulated the variation in the resident community in association with soil pH and the C/N ratio, but finally, the community established and showed resilience to subsequent inoculations. Consequently, the necessity of repeated inoculations should be reconsidered, and while the different microbial inoculants showed distinct impacts on resident microbiome succession, the communities ultimately exhibited resilience.

**IMPORTANCE** Introducing beneficial microbes to the plant-soil system is an environmentally friendly approach to improve the crop yield and soil environment. Numerous studies have attempted to reveal the impacts of inoculation on the rhizosphere microbiome. However, little is known about the effectiveness of periodic inoculations on soil functioning. In addition, the long-term impact of repeated inoculations on the native community remains unclear. Here, we track the succession traits of the resident microbiome in the bulk soil across a growing season and identify the taxon clusters that respond differently to periodic inoculation. Crucially, we compare the development of the resident community composition with and without inoculation, thus providing new insight into the interactions between resident microbes and intruders. Finally, we conclude that initial inoculation plays a more important role in influencing the whole system, and the native microbial community exhibits traits of resilience, but no resistance, to the subsequent inoculations.

## Abstract (precis)

Introducing beneficial microbes to the plant-soil system is an environmentally friendly approach to improve the crop yield and soil environment. Numerous studies have attempted to reveal the impacts of inoculation on the rhizosphere microbiome.

## INTRODUCTION

Soil microorganisms are the main drivers of soil ecosystem functioning, including the mineralization of organic matter, nutrient cycling, and resistance to soilborne diseases (1–3). However, the native soil microbial community is sensitive to exogenous disturbances due to anthropogenic activities (fertilization, pesticide application, and irrigation) and natural climate change (temperature and rainfall) (4, 5). The impacts of abiotic disturbances, such as chemical fertilization and water stress, on soil microorganisms have been widely reported (6, 7). In addition, soil-resident microbial communities are frequently subjected to biotic disturbances such as the application of biocontrol or beneficial microbial inoculants and naturally occurring microbial disturbances such as soilborne pathogens (8, 9). These invading microbes, whether beneficial microbial inoculants for promoting plant productivity or harmful pathogens affecting plant health, can alter microbial community succession, composition, and diversity (10, 11).

The host plants can assemble beneficial microorganisms in the rhizosphere via signals such as root exudates in response to attack by soilborne pathogens (12). As a manual and sustainable soil management strategy, microbial inoculants are efficient and ecofriendly for improving crop productivity and soil properties, with living beneficial microorganisms colonizing the rhizosphere and increasing nutrient availability to the host plant (13, 14). Several studies have explored the influence of one-off microbial inoculation on soil nutrients, plant growth, and defense against pathogens (15–17). However, these beneficial effects are frequently restricted due to many factors, e.g., soil nutrient (18) and organic matter (19) contents, seasonal variation (20), and competition with resident microbiota (21). To achieve sustained benefits on soil properties and plant growth, periodic applications of microbial inoculants might be helpful. However, not all invasive microbes can successfully join the resident community; soil resources and the composition of the native community determine resilience and resistance to intruders (22).

Disturbances are often classified as pulse (short term) or press (continuous or long term) depending on their duration and influence on the soil properties (23). Although beneficial microbial inoculants can be effective remediation agents in soil, successive inoculation may act as a press disturbance that directly or indirectly disrupts the native soil microbial habitat (11, 24). Press disturbances of soil microbial communities due to long-term inorganic or organic fertilization have been reported for a wide range of locations and crop types (25–27), but little information is available on the response of the soil-resident microbial community to repeated inoculant inputs. Previous studies (9, 28) suggested that a single microbial invasion may alter the resident community composition, functioning, as well as nutrient niche breadth and that microbial diversity determines the outcome of biotic invasions, but the extent and persistence of the influence of periodic microbial inoculations on shifts in native communities remain unclear. P. C. Mawarda et al. (29) also indicated that the deliberate release of microbial inoculants may cause resource competition, synergism, and antagonism effects on the resident microbiome. Given the growing use of such practices, it is important to understand the underlying mechanisms of the responses of the microbial community under different inoculant additions in order to evaluate soil quality and resilience (30).

The influences of different microbial inoculants on soil properties under controlled conditions and the practical effects on plant nutrient uptake under natural conditions have been thoroughly evaluated (31–33). In this study, we sought to investigate the dynamics of soil nutrients, plant growth, and the soil-resident bacterial community in response to successive microbial inoculations over the course of a growing season. We hypothesized that inoculations would increase soil nutrient availability as well as plant growth and that these beneficial effects would increase along with repeated applications. We hypothesized that repeated inoculations would act as press disturbances, affect the stability of soil-resident microbes, and modulate the composition of the soil-resident microbiome. These disturbances would lead to different patterns of bacterial community shifts. Moreover, we hypothesized that different inoculants could be associated with disparate impacts on the resident microbiome, host plant growth, and soil function.

The present experiment was conducted from November 2017 to October 2018 in pots planted with the native medicinal plant Cyclocarya paliurus (Batal.) Iljinsk (34). Four plant-beneficial strains were applied alone or in combination four times with an interval of 45 days. An afforestation experiment was subsequently established in 2019 using the same inoculated seedlings to evaluate the following effects of past microbial inoculations on plant growth at a different site. The plants and bulk soils were dynamically sampled throughout the study period to (i) investigate the soil functioning and dynamic growth of plants under different inoculant types and different time points, (ii) evaluate the shifts in the native microbial community in response to periodic inoculations, (iii) identify the changing patterns of microbial taxa and the differences between different inoculation types, and (iv) analyze the underlying biotic and abiotic factors shaping the soil microbial community.

## RESULTS

### Effects of microbial inoculants on dynamic growth of *Cyclocarya paliurus*.

**FIG 1:**
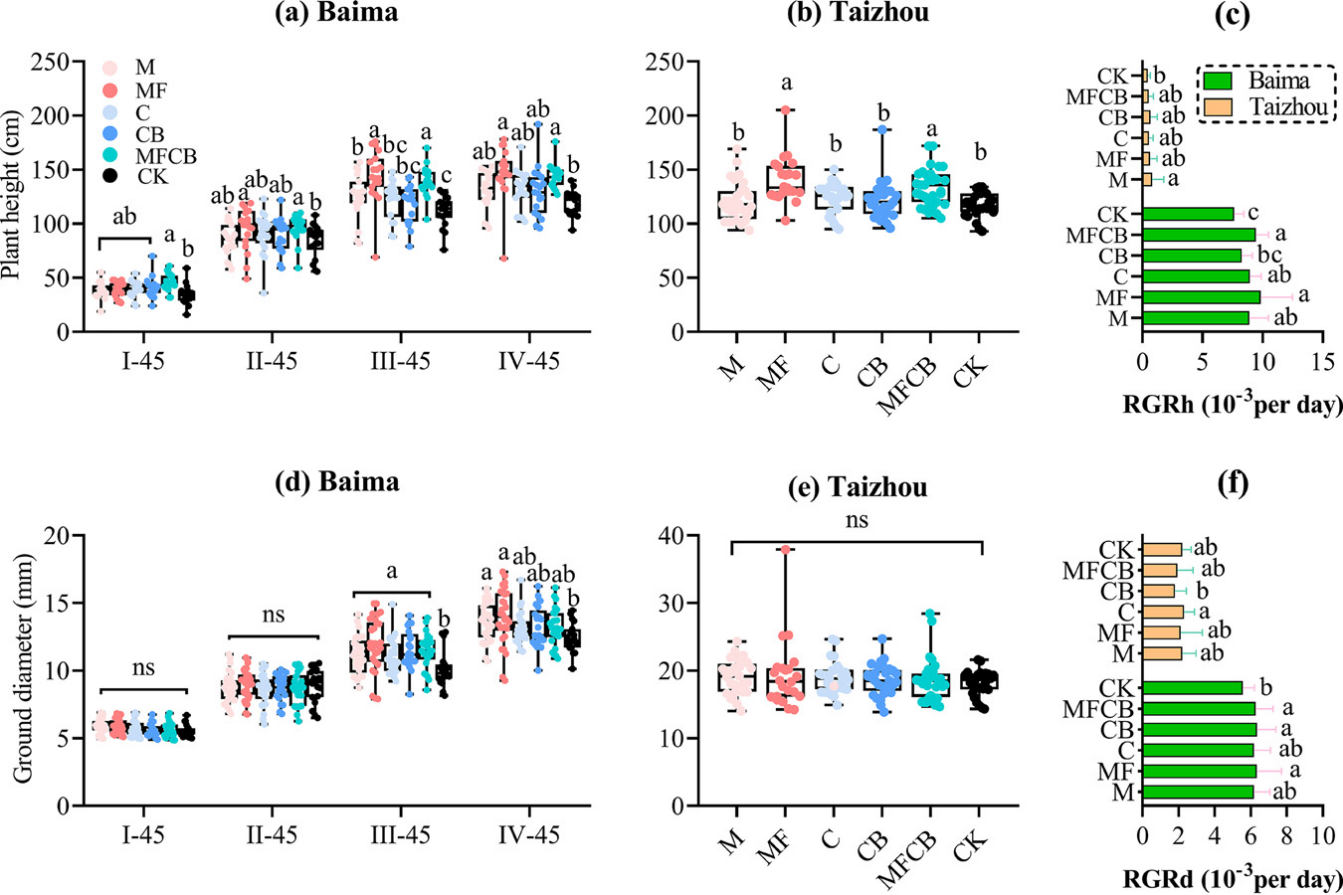
Dynamic growth of *C. paliurus* height (a) and ground diameter (d) in Baima (inoculation period), final height (b) and ground diameter (e) of *C. paliurus* in Taizhou (transplantation period), and relative growth rates of height (c) and ground diameter (f) in Baima and Taizhou. The sampling days were I-, II-, III-, and IV-45 (45 days after the first, second, third, and fourth inoculations, respectively). The treatments were M or C (single application of Bacillus megaterium or Azotobacter chroococcum, respectively), MF (dual application of B. megaterium and Pseudomonas fluorescens), CB (dual application of *A. chroococcum* and Azospirillum brasilense), MFCB (application of all four strains), and CK (noninoculation).

The growth indices of C. paliurus were dynamically measured during the inoculation period (Baima, Nanjing, China) and the transplantation period (Taizhou, China). In Baima, only MFCB (Bacillus megaterium W17 [M], Pseudomonas fluorescens W12 [F], Azotobacter chroococcum HKN-5 (C), and Azospirillum brasilense [B]) treatment significantly increased the seedling height 45 days after the first inoculation, while no significant improvement of ground diameter was found during this period. After the second inoculations, we observed improved plant height growth for treatments containing Bacillus megaterium and Pseudomonas fluorescens, i.e., MF and MFCB (Fig. 1a and d), but no significant effects were found in other treatments. In Taizhou, significant increases in plant height were observed in treatments MF and MFCB, but the differences in ground diameter between inoculated and noninoculated seedlings were not significant. In terms of relative growth rates of height (RGRh) and ground diameter (RGRd), inoculations, especially MF, CB, and MFCB, increased the RGRh and RGRd of *C. paliurus* in Baima, while a very limited impact of microbial inoculation was observed during the transplantation period (Fig. 1c and f). Statistical results by Student’s *t* test indicated that the differences between each treatment in Baima and Taizhou were significant (*P < *0.05).

### Soil biochemical properties.

During the inoculation period, soils were collected at six time points (I-10 [10 days after the first inoculation {inoculation I}], I-30, I-45, II-45, III-45, and IV-45) to determine soil biochemical properties. According to the two-way analysis of variance (ANOVA) results (see Fig. S1 in the supplemental material), the factors time (varied from *P *< 0.0001 to *P* = 0.0339) and inoculant type (varied from *P* < 0.0001 to *P *= 0.0687) played key roles in explaining the variation of biochemical properties, but their interaction was not significant (*P > *0.1) for explaining the variations in soil pH and the C/N ratio. After the first inoculation, soil available nutrients differed significantly between inoculated soils and the control during the first 10 to 90 days. However, the impacts of inoculation lessened over the period of 45 to 90 days, and the only significant differences were increases in available phosphorus (SAP) and soil alkali-hydrolyzable nitrogen (SAN) contents in treatments of MFCB and CB, respectively. The soil pH was lower in the first 10 days and the last 90 days than with the control (*P < *0.05) (Fig. S1e and f). Inoculation time significantly influenced soil nitrogenase activity and acid phosphatase activity, but the patterns of change differed. Soil nitrogenase activity decreased 45 days after the first inoculation (I-45) and recovered after the second and third inoculations. In contrast, phosphatase activity showed an increasing trend over the first 180 days, and a significant dependence of activity on the inoculation time was also observed.

### Bacterial diversity based upon 16S rRNA gene sequencing.

After subsampling each of the total of 115 samples to an equal sequencing depth, a total of 10,978 operational taxonomic units (OTUs) at 97% identity were obtained, with a range of 1,952 to 2,932 OTUs per sample. According to Good’s coverage estimator (with an average of 97%) (Table S1), nearly complete sampling of bacterial community diversity was obtained for all treatments. Compared with the OTU numbers at the time before inoculation (2,607) (data not shown), the observed OTUs significantly increased at I-10 and I-30, but little effect of treatment was observed (Table S2). Inoculation had no effect on the Shannon and Simpson indices after I-45, whereas the ACE (abundance-based coverage estimators) and Chao1 indices were significantly impacted by inoculation during the first 45 days. The effects of the different microbial consortia varied in the initial period; inoculation with four strains (MFCB) and two N_2_-fixing bacteria (NFB) (CB) increased Simpson values at I-10, whereas the ACE index was lower in the treatments with phosphate-solubilizing bacteria (PSB) (M and MF) than with the noninoculation treatment (CK) (*P* < 0.05). According to the overall ANOVA results (Table S3), sampling day significantly affected bacterial diversity and richness, but no significant effects of treatments or their interactions on bacterial diversity indices were observed across the entire study period.

### Shifts of resident bacterial community composition under repeated microbial inoculations.

The relative abundances of the top 11 phyla represented ∼96% of the total communities (Fig. S2). Most of the bacterial sequences obtained from our experimental soils belonged to the phyla *Proteobacteria* (42 to 54%), *Bacteroidetes* (5 to 10%), *Actinobacteria* (5%), and *Acidobacteria* (5 to 21%); the remainder (16 to 20%) belonged to the phyla *Firmicutes*, *Chloroflexi*, *Gemmatimonadetes*, *Verrucomicrobia*, *Planctomycetes*, and *Armatimonadetes*.

**FIG 2:**
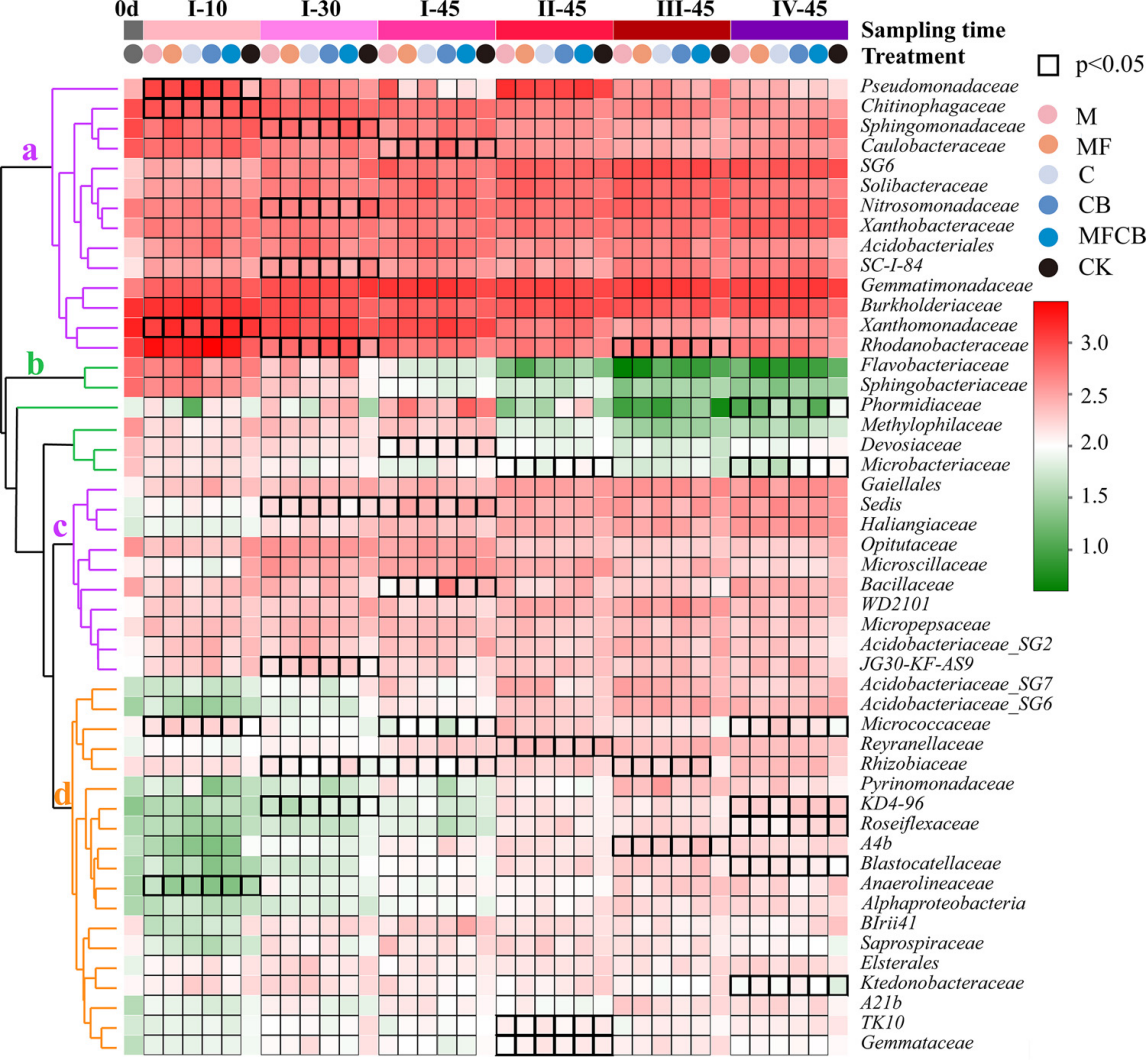
Heat map of the bacterial community at the family level (top 50) under periodic inoculations over time. Black boxes indicate the statistical significance of differences between treatments at each time point. a, b, and c show different changing patterns of bacterial taxa across all sampling time points clustered based on abundance similarities between taxa. The sampling days were 0d (the day before microbial inoculation); I-10, I-30, and I-45 (10 days, 30 days, and 45 days after the first inoculation, respectively); and II-, III-, and IV-45 (45 days after the second, third, and fourth inoculations, respectively). The treatments were M or C (single application of Bacillus megaterium or Azotobacter chroococcum, respectively), MF (dual application of B. megaterium and Pseudomonas fluorescens), CB (dual application of *A. chroococcum* and Azospirillum brasilense), MFCB (application of all four strains), and CK (noninoculation).

The bacterial community composition at the phylum level varied significantly across the different sampling times (180 days), with less pronounced effects of inoculation treatment (Table S4). However, there were significant differences in families between treatments, as shown in Fig. 2 (*P* < 0.05). In response to periodic inoculations, the temporal variation of the top 50 families exhibited three distinct patterns with respect to time: resilience (patterns a and c), antagonism (b), and synergism (d) (Fig. 2). It should be noted that the significant differences between treatments were mostly found within the first 45 days after the first inoculation (I-10, I-30, and I-45) in pattern a. In this period (0 to 45 days), the relative abundances of families like *Pseudomonadaceae* and *Micrococcaceae* in all treatments, *Xanthomonadaceae* in MF and MFCB, and *Rhodanobacteraceae* in CB and MFCB significantly increased (*P* < 0.05) compared to the control. The family *Chitinophagaceae* decreased in the CB treatment, and *Anaerolineaceae* significantly decreased in all treatments (*P* < 0.05). However, after 45 days, the bacterial community in pattern a exhibited resilience to the following disturbances, and no significant differences were found between inoculated and noninoculated soils.

### Effects of repeated microbial inoculations on overall bacterial community structure.

**FIG 3:**
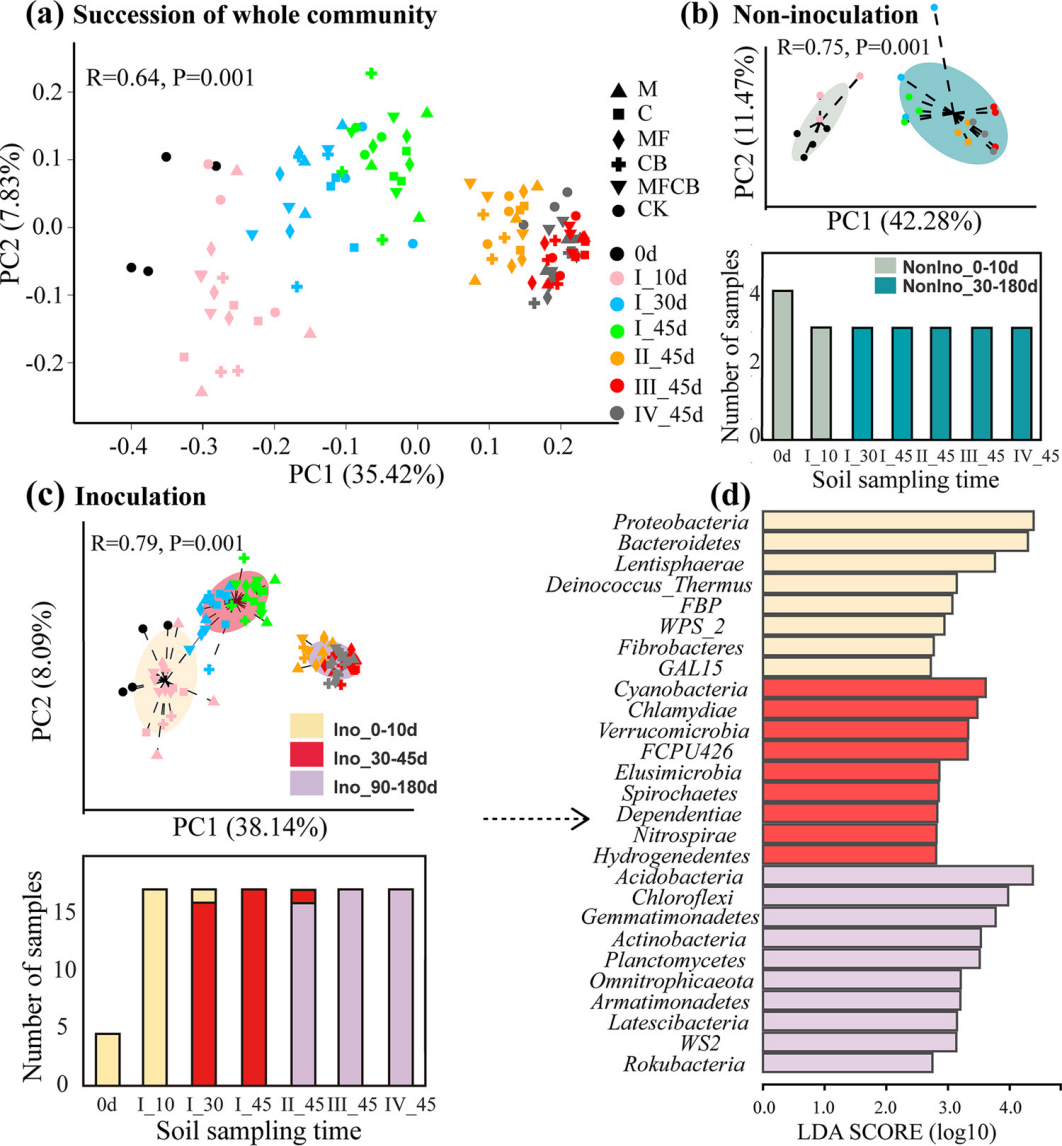
Temporal variation of bacterial community structure under different soil managements. (a) Succession of the resident soil bacterial community as revealed by principal coordinates of Bray-Curtis similarities. (b) Bacterial community clusters (PCoA plot) and their dominations (bar plot) in the succession of noninoculated soils across all time points. NonIno_0-10d and NonIno_30-180d indicate two main clusters for noninoculated samples as derived from community type analysis. (c) Bacterial community clusters and their dominations in the succession of inoculated soils across all time points. Ino_0-10d, Ino_30-45d, and Ino_90-180d indicate three main clusters for inoculated samples as derived from community type analysis. (d) Differences in phylum abundances among the three clusters found in the inoculated soils according to linear discriminant analysis (LDA) scores. The sampling days were 0d (the day before inoculation); I-10, I-30, and I-45 (10 days, 30 days, and 45 days after the first inoculation, respectively); and II-, III-, and IV-45 (45 days after the second, third, and fourth inoculations, respectively). The treatments were M or C (single application of Bacillus megaterium or Azotobacter chroococcum, respectively), MF (dual application of B. megaterium and Pseudomonas fluorescens), CB (dual application of *A. chroococcum* and Azospirillum brasilense), MFCB (application of all four strains), and CK (noninoculation).

Principal-coordinate analysis (PCoA) ordination based on Bray-Curtis dissimilarities at the OTU level indicated the succession of the soil bacterial community over the course of the experiment (Fig. 3a). In accordance with the results of community composition, the community changed significantly in the first 45 days (I-10, I-30, and I-45) (*R*^2^ = 0.24 and *P* = 0.001 by permutational multivariate analysis of variance [PERAMONA]), but the community dissimilarities within the last three time points decreased. The pairwise correlations between different time points also indicated that the whole microbiome stabilized at the last three time points (Fig. S3).

To further examine the differences between inoculated and noninoculated soils over time, typing analysis was conducted based on the Bray-Curtis dissimilarity in the PCoA plot (Fig. 3b and c). At all seven time points (including the day before inoculation [0d]), three bacterial cluster types were found in inoculated soil, whereas only two bacterial cluster types were detected in the control (*R*^2^ = 0.40 and *P* = 0.01 by PERAMONA for five types). Bar plots (Fig. 3b and c) were used to depict the compositions of these cluster types at each time point, showing that repeated inoculations altered the community succession compared to noninoculated treatment (Fig. 3b). It took approximately 10 to 30 days for the bacterial community in noninoculated soil to change from NonIno_0-10d (the community cluster in noninoculated samples during the first 10 days) to NonIno_30-180d (Fig. 3b). The bacterial community in the inoculated soil also completed this change from Ino_0-10d (the cluster in the inoculated samples during the first 10 days) to Ino_30-45d, but after the second inoculation, Ino_30-45d was transformed into Ino_90-180d and remained stable thereafter (Fig. 3c). To illustrate the dynamics of community composition and compare the differences between different cluster types, we identified the OTUs in different types and visualized community succession on the phylum level (Fig. S4). The *Acidobacteria* phylum significantly increased in inoculated samples but stayed stable in noninoculated soil. On the contrary, the *Bacteroidetes* phylum decreased over time in inoculated samples but increased in noninoculated samples.

**FIG 4:**
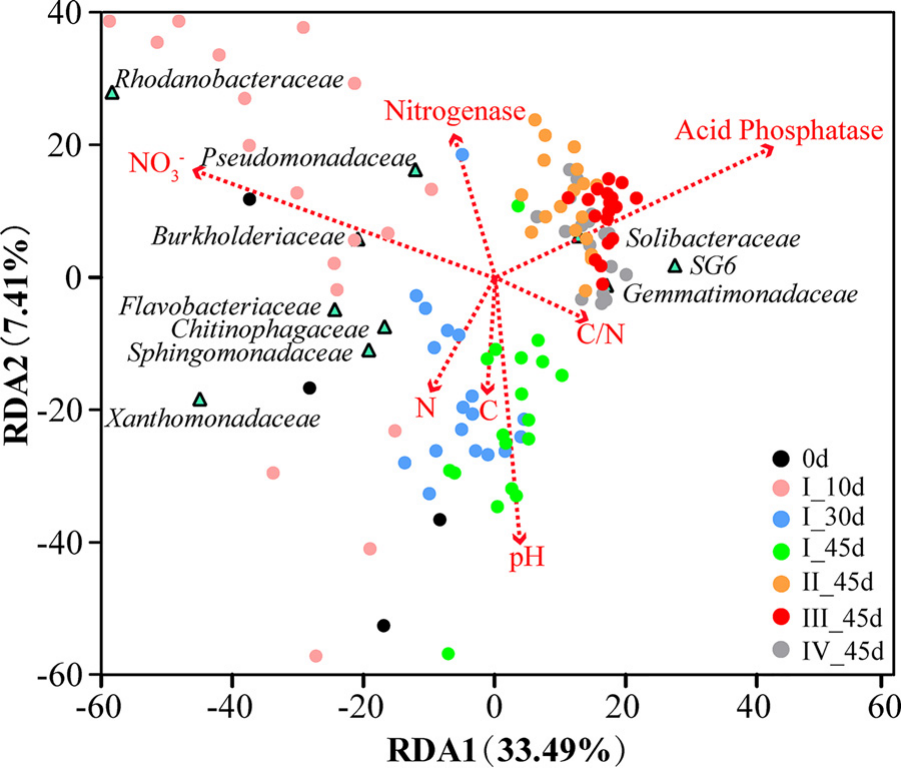
Redundancy analysis illustrating the effects of environmental factors on the succession of the bacterial community and top 10 families across all treatments. The sampling days were 0d (the day before inoculation); I-10, I-30, and I-45 (10 days, 30 days, and 45 days after the first inoculation, respectively); and II-, III-, and IV-45 (45 days after the second, third, and fourth inoculations, respectively).

Linear discriminant analysis (LDA) revealed differences in phylum abundances among the three cluster types (Ino_0-10d, Ino_30-45d, and Ino_90-180d) found in inoculated soil (Fig. 3d). The top 3 markers based on LDA scores were *Proteobacteria*, *Bacteroidetes*, and *Lentisphaerae* for Ino_0-10d; *Cyanobacteria*, *Chlamydiae*, and *Verrucomicrobia* for Ino_30-45d; and *Acidobacteria*, *Chloroflexi*, and *Gemmatimonadetes* for Ino_90-180d. Soil properties (C, N, S, C/N ratio, nitrate, pH, and enzyme activity) were examined for their abilities to explain the bacterial community variation in inoculated soils (Fig. 4). Among these factors, nitrate and acid phosphatase activities explained 46.1% and 42.3% of the bacterial community variation along axis 1, respectively, and soil pH explained the most variation along axis 2 (39.1%).

### The route of community change is transiently modulated by single or mixed microbial inoculants.

**FIG 5:**
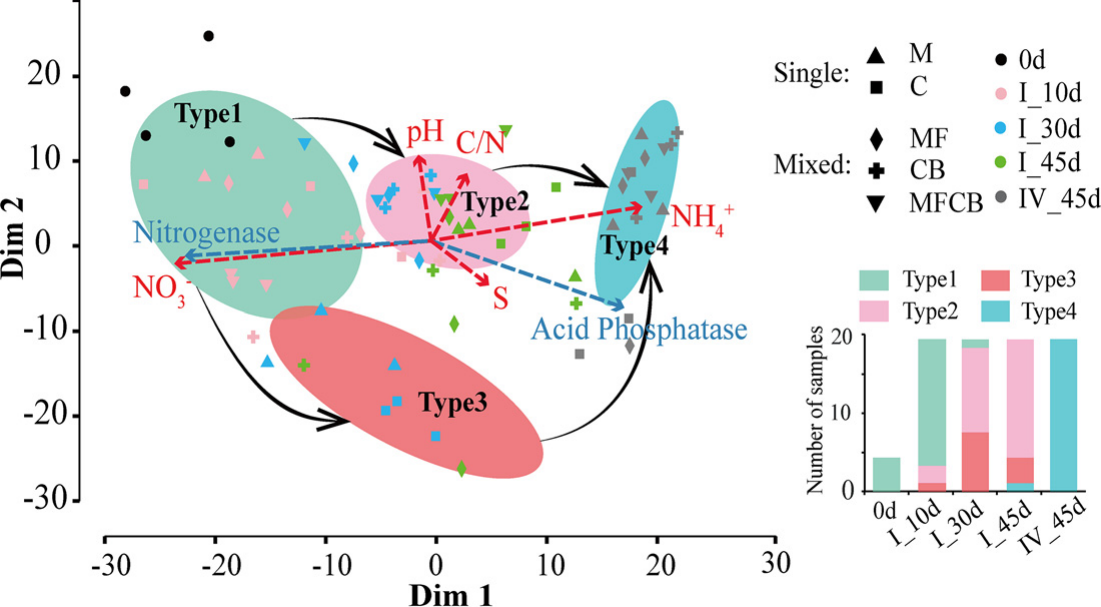
Typing analysis of the temporal variation of bacterial community structure under different inoculations at I-10, I-30, I-45, and IV-45. Different routes from type 1 to type 4 were identified: type 1-2-4 (for treatments with MF, CB, and MFCB) or type 1-3-4 (for treatments with M and C). The column diagram indicates a single-complex-single pattern of change in the presence of the four cluster types at the five sampling time points. The sampling days were 0d (the day before inoculation); I-10, I-30, and I-45 (10 days, 30 days, and 45 days after the first inoculation, respectively); and IV-45 (45 days after the fourth inoculation). The treatments were M or C (single application of Bacillus megaterium or Azotobacter chroococcum, respectively), MF (dual application of B. megaterium and Pseudomonas fluorescens), CB (dual application of *A. chroococcum* and Azospirillum brasilense), and MFCB (application of all four strains).

Since the dissimilarities in the bacterial community at II-45, III-45, and IV-45 were smaller than those in the first 45 days, we selected five time points in the first 45 days and the last 45 days (Fig. 5) to evaluate the different effects of the inoculant types on the soil-resident bacterial community. Four cluster types (types 1 to 4) were obtained from these samples across these five time points, and the routes of community change from type 1 to type 4 differed according to treatments. The route was type 1-2-4 for inoculation with mixed strains (MF, CB, and MFCB) but type 1-3-4 for the single-strain treatments (M and C) (Fig. 5). In addition, across all five selected time points, a single-complex-single cluster pattern was observed (Fig. 5, stacked-column plot). These patterns suggest that the microbial inoculants modulated different subsets of the microbial community in soil for a short period, even though all inoculants ultimately resulted in similar clustering patterns.

Soil factors were analyzed to identify potential abiotic parameters affecting the succession of the resident microbial community over time (Fig. 5). Inorganic N (nitrate and ammonium) and the activities of nitrogenase and acid phosphatase were the main factors driving the temporal variations of microbial community structure, whereas soil pH and the C/N ratio, followed by nitrate, were the main factors explaining the difference between the single- and mixed-inoculant treatments at I-30. To explore the biological factors underlying the microbial community differences between the single- and mixed-inoculant treatments, we further compared taxonomic markers from the order to the family levels at I-30 based on linear discriminant analysis effect size (LEfSe) (Fig. S5). The top 3 markers in soils inoculated with mixed strains were *Xanthomonadales*, *Sphingomonadales*, and *Sphingomonadaceae*, whereas *Solibacteraceae*, *Solibacterales*, and *Thermoanaerobaculia* were the top 3 taxa in single-strain-inoculated soil.

## DISCUSSION

### Responses of plant growth and soil functioning to repeated inoculations.

Soil-beneficial microorganisms interact intimately with the roots of the host plant and affect the ecological adaptability of the plant to its environment. Nonetheless, these beneficial effects can be weakened by intensive land usage, thereby decreasing the plant’s capacity to deal with biotic and abiotic stresses (35). Consequently, we hypothesized that the beneficial effects of microbial inoculants on soil nutrients and plant growth would increase with repeated applications. However, different from our hypothesis, we found that periodic inoculations mostly increased soil available nutrients during the first 10 to 90 days. Although the advantages of treatments with MFCB and CB appeared to be pronounced at the last two sampling times, the benefits of inoculation generally decreased over time. This indicates that the effects of microbial inoculants on bulk soil functioning were transient rather than persistent during the investigation. This is similar to a previous study in which the inoculated strain caused no major changes in rhizosphere community function (36). It should be noted that the changes in the bulk soil might be different from those in the rhizosphere soil, and these changes can also in turn affect the microbial community (37). Nonetheless, in the present study, PCoA and community type analysis confirmed that the resident bacterial community in the bulk soil underwent shifts in the first 90 days but showed resilience to the subsequent inoculations in the last 90 days. This is in accordance with the observed variation of nitrogenase activity and soil inorganic nitrogen content. Given the resilience and resistance of the resident microbiome (38), we speculate that this decrease could be due to changes in, or the stability of, the soil microbial community. In addition, the decrease in soil nutrient content at the last time points may also be due to seasonal variation and nutrient uptake by plants. Our previous study confirmed that these microbial inoculants enhanced nutrient uptake and stimulated plant growth and biomass accumulation after whole-inoculation procedures (32).

Even though introduced microbial inoculants sometimes cannot compete efficiently with native microbial communities in soil, they stimulate root growth and modify plant metabolism at very early stages and might generate lasting effects on the root system and associated microbial communities (39). In the present study, microbial inoculations significantly promoted *C. paliurus* growth and reshaped root morphological traits (more fine roots and lateral roots in the inoculated seedlings [data not shown]) compared to noninoculated seedlings after the inoculation period in Baima. However, the growth-promoting effect was highly variable across time and inocula and not maintained when the seedlings were transplanted to Taizhou. The subsequent growth-promoting effects of microbial inoculants on plants might be compromised due to the ceased inoculation, thus presenting the importance of continuous microbial inoculation when transplanting and establishing plantation in a different site. Another reason could be the change of the soil environment, because plants exhibit less reliance on the soil-beneficial microbes when experiencing a normal/high-level nutrient environment and thus benefit less from the previous inoculation (40). Even though we cannot precisely track the establishment of the introduced strains in a different site, the results proved that the benefits from inoculation could decrease without subsequent applications.

### Inoculation times and types affect the composition and succession of the resident bacterial community.

Both natural and anthropogenic microbial invasions frequently start with a dominating microbial population and leave a footprint on the native soil microbiome, even though the introduced populations may decrease at last (22, 28). With the increasing demand for biofertilizers in agroecosystems, the question of whether repeated application of biofertilizer (such as beneficial microbial inoculants) influences the resident soil community warrants investigation. In addition, with regard to the introduction of plant-growth-promoting rhizobacteria (PGPR), previous studies (41, 42) have attempted to evaluate the impacts on the microbial community in the rhizosphere, while less is focused on the changes of the bulk soil community. To address these questions, we evaluated to what extent and how long the repeated applications of inoculants (not native) impacted the dynamics of the resident bacterial community in the bulk soil. In response to repeated inoculations, three patterns (fold increase, fold decrease, and resilience) of shifts in bacterial composition were observed; 57% of the significant variation among treatments occurred during the first 45 days. Changes in soil nutrients were consistent with these shifts. Furthermore, microbial inoculants may alter the resident community composition by causing resource competition, synergistic effects, and antagonistic effects (29). In the present study, the relative abundances of families like *Xanthomonadaceae* significantly increased in the treatments with PSB, suggesting that the introduction of PSB facilitated specific resident populations, which is in accordance with a previous study (37). In contrast, *Chitinophagaceae* and *Rhodanobacteraceae* significantly decreased in soil inoculated with NFB. These declines in the abundances of some taxa after the initial disturbance due to microbial inoculation may be a result of competition for similar preferred niches and available resources in the soil (28, 43).

The resident soil bacterial community exhibited a high level of resilience, but not resistance, to the microbial disturbance caused by periodic inoculations. The initial inoculation disturbed the stability of the resident microbiome, which was as a result more susceptible to subsequent inoculation disturbances. This is in line with the above discussion that the effects of such amendments both below- and aboveground are transient. PCoA, community type analysis, and pairwise correlation analysis confirmed that the dissimilarity between the communities decreased in the last 90 days. This suggests resilience of the resident microbiome upon repeated inoculation disturbances, similar to other reports of resilience within soil microbial communities (11, 44). Surprisingly, in the present study, the second inoculation still left a footprint on the resident community, resulting in an increase in the number of cluster types in the inoculated soils (Ino_0-10d, Ino_30-45d, and Ino_90-180d) compared with the control (NonIno_0-10d and NonIno_30-180d). This finding also confirms the previously proposed hypothesis that a second disturbance by the same invader could persist longer or even naturalize into the community (28). It should be mentioned that we did not use specific primers to track the persistence of inoculated strains in soil; however, 16S rRNA gene sequencing showed that the introduction of microbial inoculants altered the seasonal succession of the resident community. The unexpectedly strong impact of soil management over temporal effects on the resident community is supported by previous observations in different agricultural systems (5, 45), but this study revealed the relationship between repeated inoculations and the native microbiome. Although this work provided detailed information about how the inoculation period and type affect the resident microbes, future studies should consider setting a unique control that receives only one dose at first and is sampled at the end of the experiment to further compare the influences of repeated inoculation to those of one-off inoculation. Furthermore, both insignificant (46) and significant (47) effects on the native microbial community structure were observed in the rhizosphere soils after PGPR inoculation. Hence, it would be interesting for future studies to compare the differences in community succession in bulk soil and rhizosphere soil.

### Underlying factors shaping the resident microbiome during the application of microbial inoculants.

The changes in soil chemical factors due to beneficial microbial inoculation, such as nitrate and pH, were the dominant factors explaining the succession of the resident community over time. Kuramae et al. (48) also reported that soil pH significantly altered the trajectory of microbial secondary succession. Confirming this result, after the first and the fourth inoculations, the soil pH in inoculated treatments significantly differed from that in noninoculated soil. The PSB possess the ability to produce organic acid during the decomposition of soil organic matter, which is associated with the release of P from mineral-bound complexes such as AlPO_4_ and FePO_4_, thus leading to a decrease of the soil pH and changes in the related nutrient contents (49). On the other hand, NFB are able to increase the contents of ammonium and consequently improve nitrites with the help of nitrifying bacteria. In the present study, the contents of inorganic N after the first inoculation were significantly increased compared to the control. To evaluate the potential impacts of the growth medium on the changes in soil properties and plant performance, we confirmed that the addition of bacterial growth medium exhibited no significant impacts on plant growth, biomass, and nutrient acquisition and showed a very limited influence on soil available nutrients (32). However, it cannot be ruled out that other factors not assessed in this study might be driving this seasonal variation.

For the identified taxonomic markers for each cluster in the inoculated soils, the phyla *Proteobacteria* and *Bacteroidetes* generally have copiotrophic strategies with rapid growth responses to resource availability (50). In this study, these phyla were enhanced during the first 45 days after inoculation, which is also the period for the rapid change in soil nutrients. *Cyanobacteria* are emerging beneficial microorganisms with the ability to control nitrogen deficiency and sensitivity to fertilization (51, 52), whereas *Chlamydiae* and *Verrucomicrobia* are sensitive to soil moisture and time (seasonal variation) (53, 54). These phyla were significantly more abundant in cluster Ino_30-45d than in the other cluster types, indicating contributions of both microbial inoculation and seasonal variation. The presence of *Acidobacteria* in cluster Ino_90-180d is likely attributable to the low soil pH at the last sampling time compared with the control, which seems to favor this bacterial phylum (55). The phyla *Chloroflexi* and *Gemmatimonadetes* are widely known to be enriched in dry-season soil (56, 57). Overall, the formation of different cluster types is likely attributable to both seasonal variation and changes in soil biochemical properties caused by periodic inoculations.

Mixed inoculants of different strains have been widely developed and evaluated for their great potential in enhancing plant growth and soil nutrients (58–60). In this study, plant growth exhibited a strong preference for the mixed inoculants MFCB, which presented the highest growth of height and ground diameter during the whole inoculation period. Dual inoculants such as MF also showed significant advantages compared to the single inoculant M in improving soil enzyme activities at certain time points. It has been proposed that coinoculation permits synergistic interactions that stimulate physical or biochemical activities and simultaneously improve microbial viability (60), thus bringing more interaction with the soil and host plant such as the production of enzymes and organic acid. On the other hand, coinoculation may leave a different footprint on the resident microbiome than single inoculation because more ecological niches would be required for mixed inoculants than when these organisms are used alone (60–62). In addition, the nature of such differences could also due to the feedback of changed soil environments and plant performance. In the present study, different inoculants (single/mixed) transiently modulated the variation of the resident community 30 days after the first inoculation. Soil pH and the C/N ratio were the main factors underlying this impact, followed by nitrate. Confirming this result, the soil C/N ratio at I-30 was higher in single inoculants than in mixed inoculants. However, the difference in pH between the single- and mixed-inoculant treatments was not significant. Hence, other environmental factors that were not assessed in this study could be driving these differences. For the biotic factors, bacterial taxa like *Solibacteraceae*, *Solibacterales*, and *Thermoanaerobaculia* (all belonging to the phylum *Acidobacteria*) were identified as markers for the single treatments based on LDA scores. The abundance of the phylum *Acidobacteria* is closely related to soil pH and resources such as total nitrogen and nitrate (55, 63, 64), being consistent with the soil factors discussed above. It should be noted that the succession difference of resident communities derived from single and mixed inoculants was observed for only a short period; the resident community established and behaved similarly at last.

In conclusion, repeated inoculations did not ideally improve the benefits from microbial inoculants, and the beneficial effects on plant growth were not maintained after transplantation to a different site. Consequently, the necessity of repeated microbial inoculations should be reconsidered. The resident bacterial community in bulk soil exhibited traits of resilience, but not resistance, to repeated inoculation. This study revealed that the changes in the resident community mostly reflected the initial disturbance of inoculant addition and partially explained the variations in soil nutrients and subsequent plant growth. The responses of bacterial taxa in the soil to microbial inoculants depended on the inoculant types (PSB or NFB) and taxon clusters. In response to periodically introduced microbes, resilient changing patterns included the main taxa of the resident microbiome. Inoculation and noninoculation significantly differed during the succession of the community and resulted in different cluster types and composition shifts, thus providing new insight into understanding the interactions between resident microbes and intruders. Soil pH and nitrate were the main factors explaining the succession of the resident community, leading to the development of three cluster types over time. The single and mixed inoculants briefly modulated the variation of the resident community in association with soil pH and the C/N ratio. However, over time, bacterial communities established and showed a high level of resilience.

## MATERIALS AND METHODS

### Site description and material preparation.

The seedling nursery site was a semiautomatic plant growth unit located in Baima, Nanjing, China (31°35′N, 119°10′E), while the afforestation site was located at the Jiangsu Traditional Chinese Medicine (TCM) Science and Technology Park, Taizhou, China (32°37′N, 119°98′E). These sites (115 km apart) are in the typical transition zone from the north subtropics to the subtropics and have the same soil type (clay loam soil), abundant rainfall (1,037 mm/year) and sunshine (2,146 h/year), and an annual average temperature of approximately 15.4°C. The soil properties of Baima are pH 5.98, total C of 18.9 g·kg^−1^, total N of 1.61 g·kg^−1^, total P of 0.42 g·kg^−1^, available N of 12.68 mg·kg^−1^, and available P of 5.56 mg·kg^−1^, whereas in Taizhou, the soil properties are pH 7.31, total C of 12.72 g·kg^−1^, total N of 0.88 g·kg^−1^, total P of 0.45 g·kg^−1^, available N of 88.35 mg·kg^−1^, and available P of 32.22 mg·kg^−1^.

Four beneficial strains, Bacillus megaterium W17, Pseudomonas fluorescens W12, Azotobacter chroococcum HKN-5, and Azospirillum brasilense CW903, were used alone or in combination in this study. Our previous study monitored the effects of single and mixed inoculants on soil properties and their survival dynamics in the soil (31), thus providing a reference for selecting the appropriate microbial inoculants and inoculation period for this study. According to their survival abilities and effects on soil, we selected single inoculants (M, inoculation with B. megaterium; C, inoculation with A. chroococcum) and mixed inoculants (MF, inoculation with both B. megaterium and P. fluorescens; CB, inoculation with both *A. chroococcum* and A. brasilense; MFCB, coinoculation with all four strains). These bacteria have been documented to improve soil nutrient status and do not have antagonistic effects on one another (31). Each strain was grown in lysogeny broth medium at 28°C with shaking at 180 rpm for 24 to 26 h until an optical density at 600 nm (OD_600_) of 0.9 was reached, which corresponded to the log phase. The bacterial population was examined in a laboratory using the plate count serial dilution method while experimenting on building a standard curve between optical densities and bacterial quantities. The suspensions were adjusted to a final concentration of 1 × 10^8^ CFU ml^−1^ for each strain based on the OD_600_.

### Experimental design and soil sampling.

**FIG 6:**
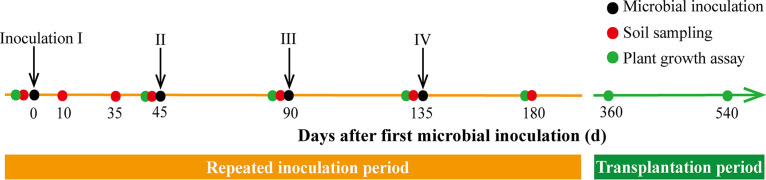
Timeline for microbial inoculation, soil sampling, and plant growth measurement. The major part of the experiment was conducted in 2018 in Nanjing (inoculation period). After that, seedlings were transplanted to Taizhou in 2019 (transplantation period).

The pot experiment was laid out in a three-block pattern based on a randomized complete block design with five inoculant types (M, MF, C, CB, and MFCB). The noninoculated samples served as controls in this study because our previous study indicated that the single addition of growth medium did not significantly impact plant growth, biomass, and nutrient acquisition compared to noninoculated samples (32). Each treatment consisted of 60 *C. paliurus* seedlings that were equally divided into three blocks. The container seedlings were transplanted to the pots on 1 November 2017, and each seedling pot (top diameter, 25 cm; bottom diameter, 20 cm; height, 30 cm) contained 5 kg soil as the growth medium. Inoculations were conducted four times with an interval of approximately 45 days (4 April, 19 May, 6 July, and 19 August 2018), with the same dose each time (5 × 10^9^ cells per plant) (Fig. 6). Briefly, we dug a 5-cm-deep circle around the pot (near the edge of the plant roots) for all seedlings (including CK) to access the lateral root. Next, 50 ml of the inoculum was injected into each circle, which was subsequently covered by soil. After that, all inoculated seedlings, including pot soils, were transplanted to a different site (Taizhou) in March 2019 with the same experimental design to evaluate the legacy effects of past inoculations on plant growth.

For each treatment in each block, five bulk soil samples (0 to 10 cm) were randomly collected and equally mixed into one sample, resulting in a total of three samples for each treatment in three blocks. The sampling method was described previously (31). Briefly, five to eight random vertical holes (diameter, 8 mm; depth, 60 mm) were implemented with a sampling tube for each pot to lessen the disturbance of sampling on microbes; this provided about 50 g soil for each duplicate of each treatment. The sampling times were the day before the first inoculation (0d), 10 days after the first inoculation (I-10), 30 days after the first inoculation (I-30), 45 days after the first inoculation (I-45), 45 days after the second inoculation (II-45), 45 days after the third inoculation (III-45), and 45 days after the fourth inoculation (IV-45) (Fig. 6). The bulk soil samples were split into two parts: one was stored at 4°C prior to the analysis of biochemical properties, and the other was stored at −20°C prior to DNA extraction.

### Plant growth measurements.

Plant growth was evaluated as seedling height and ground diameter, which were measured for all healthy seedlings before the first inoculation and 45, 90, 135, 180, 360, and 540 days after the first inoculation (Fig. 6). The mean relative growth rates in height (RGRh) and ground diameter (RGRd) were also calculated as described previously by Mazarura et al. (65). The equations are as follows, where *h_i_* or *c_i_* is the initial growth in height (centimeters) or ground diameter (milliliters), *h_f_* or *d_f_* is the final height (centimeters) or ground diameter (millimeters), and *t*_2_ − *t*_1_ represents the time difference (*d*) between the initial and final sampling dates:

(1) $$\text{RGRh}=\frac{\log_{e}h_{f}-\log_{e}h_{i}}{t_{2}-t_{1}}$$

(2) $$\text{RGRd}=\frac{\log_{e}d_{f}-\log_{e}d_{i}}{t_{2}-t_{1}}$$

### Soil biochemical properties.

Soil biochemical properties included soil pH; the C/N ratio; the contents of soil alkali-hydrolyzable nitrogen (SAN), soil inorganic nitrogen (SIN), and soil available phosphorus (SAP); and the activities of phosphatases and nitrogenase. Soil pH was determined by using a pH electrode (IQ 160 pH meter; Spectrum Technologies, Inc., USA) with a soil-to-water ratio of 1:2.5. The total C and N contents were determined using an elemental analyzer (Vario Max CN; Elementar, Hanau, Germany). The SAN content was quantified according to the method of Roberts et al. (66). The SIN content (KCl-extractable NH_4_^+^ and NO_3_^−^) was analyzed by extraction with 2 M KCl in a soil-to-solution ratio of 1:5 (wt/vol) with shaking for 1 h at 200 rpm, followed by quantification using a continuous-flow analyzer (Bran+Luebbe AA3). SAP was extracted with a 1:10 (wt/vol) mixture of ammonium fluoride and hydrochloric acid and determined using the molybdenum blue method (67). Acid phosphatase activity was assessed using a method described previously by Tabatabai and Bremner (68). The soil nitrogenase activity was measured by the acetylene reduction method (69).

### DNA extraction and Illumina MiSeq sequencing.

Soil total DNA (0.5 g soil) was extracted using the NucleoSpin soil DNA kit (Macherey-Nagel GmbH & Co. KG, Düren, Germany), according to the manufacturer’s protocols. The final DNA concentration and purity were determined by using a NanoDrop 2000 UV-visible (UV-vis) spectrophotometer (Thermo Scientific, Wilmington, DE, USA), and DNA quality was checked by 1% agarose gel electrophoresis. The V4 hypervariable regions of the bacterial 16S rRNA gene were amplified with primers 515F (5′-GTGCCAGCMGCCGCGG-3′) and 907R (5′-CCGTCAATTCMTTTRAGTTT-3′) in a thermocycler PCR system (GeneAmp 9700; ABI, USA). PCR was carried out under the following conditions: an initial denaturation step at 95°C for 5 min, followed by 25 cycles of 95°C for 30 s, 56°C for 30 s, and 72°C for 90 s and a final extension step at 72°C for 7 min. PCR amplifications were performed in triplicate in a 20-μl mixture containing 4 μl of 5× FastPfu buffer, 2 μl of 2.5 mM deoxynucleoside triphosphates (dNTPs), 0.8 μl of each primer (5 μM), 0.4 μl of FastPfu polymerase, and 10 ng of template DNA. The PCR amplicons were purified using the AxyPrep DNA gel extraction kit (Axygen Biosciences, Union City, CA, USA), triplicate PCR amplifications for each sample were conducted and pooled as a PCR product, and samples were then sequenced on an Illumina MiSeq PE300 platform (Illumina, San Diego, CA, USA), according to the standard protocols of Majorbio Bio-Pharm Technology Co. Ltd. (Shanghai, China) (70).

To minimize the effects of random sequencing errors, raw fastq files were quality filtered by Trimmomatic (71) and merged by FLASH (72) with the following criteria: (i) reads were truncated at any site with an average quality score of <20 over a 50-bp sliding window; (ii) sequences whose overlap was longer than 10 bp were merged according to their overlap, with no more than a 2-bp mismatch; and (iii) sequences of each sample were separated according to barcodes (exact match) and primers (allowing a 2-nucleotide mismatch), and low-quality and ambiguous reads (sequences shorter than 150 bp) containing ambiguous bases were removed. Chimeras were identified and removed with the UCHIME algorithm (73). Operational taxonomic units (OTUs) were clustered at 97% similarity using UPARSE (v.7.1) and were declared invalid if fewer than four sequences were detected in one sample. The sampling effort was estimated by Good’s coverage (see Table S1 in the supplemental material). The Silva database (132/16S bacteria) was used with a minimum percent identity threshold of 70% for taxonomic assignment. Singletons were removed prior to further analysis. Mothur (v.1.30.2) was used to calculate bacterial α-diversity indices (Shannon, Simpson, Chao, and ACE) to estimate bacterial diversity and richness.

### Bioinformatics and statistical analyses.

Statistical analyses, including multiple comparisons for plant growth and soil nutrient variables, were performed using SPSS software (v.20.0; SPSS, Inc., USA). Two-way analysis of variance (ANOVA) was applied to analyze the effects of different inoculants and different sampling times on the plant height and ground diameter in Baima. One-way ANOVA was applied to evaluate the effects of different treatments on the plant height and ground diameter in Taizhou. Student’s *t* test was used to compare the differences of the same treatment between Baima and Taizhou. For sequence data, each sample was rarefied to 36,281 sequences before the α-diversity analyses (Table S1), which included Good’s coverage, observed OTU numbers, the ACE and Chao1 richness indices, and the Shannon and Simpson diversity indices. Analysis of similarity (ANOSIM) and permutational multivariate analysis of variance (PERMANOVA) were performed to evaluate significant differences in microbial community composition among the six inoculation treatments. Microbial community type analysis was conducted to evaluate the dynamic shifts in microbial community structure during the 180-day investigation (74). Briefly, according to the relative abundance of bacteria at the phylum level, the Jensen-Shannon distance (JSD) was calculated and clustered by partitioning around medoids (PAM), the optimal clustering *K* value was calculated by the Calinski-Harabasz (CH) index, PCoA (principal-coordinate analysis) was performed based on Bray-Curtis distances, and the coordinates were used to visualize differences in microbial community structure. The Pearson correlation coefficient was calculated by a cor() function using the microbiome data from each time point and visualized by using the corrplot package (75), and the significance level was tested by the cor.mtest() function.

Heat maps were generated based on the 50 most abundant taxa at the family level to output the dynamic shifts of the soil-resident community composition under different inoculants. The taxon clusters were conducted based on abundance similarities between each group in the vegan package. To explore the biological factors involved in the differences between the clusters derived from microbial community type analysis, we used linear discriminant analysis effect size (LEfSe) to identify taxonomic markers at the phylum level for three main clusters in inoculated samples, which was performed on the online platform of Majorbio Bio-Pharm Technology Co. Ltd. (Shanghai, China). We also identified taxonomic markers from the order to family levels for single inoculants (M and C) and mixed inoculants (MF, CB, and MFCB) 30 days after the first inoculation. Briefly, based on the normalized relative abundance of each level, the Kruskal-Wallis (KW) rank sum test was used to detect markers with significantly different abundances between the assigned taxa, and linear discriminant analysis (LDA) was performed to estimate the effect score of each marker (LDA threshold of 2). It emphasizes statistical significance, biological consistency, and effect relevance, allowing researchers to identify differentially abundant features that are also consistent with biologically meaningful categories (76). High LDA scores reflect significantly higher abundances of certain taxa. To investigate the taxon-environment relationship, we performed redundancy analysis (RDA) with the soil bacterial community for all samples, the top 10 families, and environmental factors. Environmental factors for each sampling time were selected by variance inflation factor (VIF) analysis, which was used to judge the collinearity among different factors.

### Data availability.

Raw sequences were submitted to the NCBI Sequence Read Archive (SRA) under SRA accession numbers SRR11699948 to SRR11700059, with BioProject accession number PRJNA630558.

## Supplementary Material

Supplemental file 1
