# Supplementary material for: Succession of the Resident Soil Microbial Community in Response to Periodic Inoculations
Source: Appl Environ Microbiol. 2021 Apr 13;87(9):e00046-21. doi: 10.1128/AEM.00046-21 (PMC8091015; doi:10.1128/AEM.00046-21)
Supplement: Supplemental file 1 [file AEM.00046-21-s0001.pdf]

Succession of the Resident Soil Microbial Community in Response to Periodic Inoculations

Zhikang Wang [a, b, c], Ziyun Chen [d], George A. Kowalchuk [c], Ziheng Xu [a], Xiangxiang Fu [a] #, Eiko E. Kuramae [b, c]

[a] Co-Innovation Center for Sustainable Forestry in Southern China, College of Forestry, Nanjing Forestry University, Nanjing 210037, China

**[b]** Department of Microbial Ecology, Netherlands Institute of Ecology (NIOO-KNAW), 6708 PB Wageningen, the Netherlands

[c] Ecology and biodiversity, Institute of Environmental Biology, Utrecht University, 3584 CH Utrecht, the Netherlands

[d] State Key Laboratory of Soil and Sustainable Agriculture, Institute of Soil Science, Chinese Academy of Sciences; Nanjing 210008, China

Running Head**:** Impacts of inoculations on resident microbiome

# Address correspondence to Xiangxiang Fu, xxfu@njfu.edu.cn

**Keywords:** Beneficial microorganisms, Periodic inoculation, Microbial community succession, Resident microbiome, Inoculant type, Soil remediation

**Table S1** Sample ID, sampling day, treatment, inoculant type, number and length of 16S rRNA gene sequences used in this study

| Sample_ID | Sampling_day (d) | Treatment | Inoculant_type | Reads per sample | Mean_length | Coverage[1] |
|---|---|---|---|---|---|---|
| A1 | 0 | 0d | - | 48921 | 375.67 | 0.9686 |
| A2 | | | | 39906 | 375.82 | 0.9685 |
| A3 | | | | 47382 | 375.52 | 0.9719 |
| A4 | | | | 49994 | 375.70 | 0.9734 |
| B1_1 | I_10 | M | PSB[2] | 43345 | 376.08 | 0.9673 |
| B1_2 | | | | 52355 | 375.58 | 0.9692 |
| B1_3 | | | | 48932 | 376.01 | 0.9707 |
| B2_1 | | C | NFB[3] | 56378 | 375.66 | 0.9742 |
| B2_2 | | | | 49395 | 375.70 | 0.9702 |
| B2_3 | | | | 53023 | 376.03 | 0.9674 |
| B3_1 | | MF | PSB | 52930 | 376.16 | 0.9705 |
| B3_2 | | | | 56317 | 375.65 | 0.9718 |
| B3_3 | | | | 56056 | 375.89 | 0.9680 |
| B4_1 | | CB | NFB | 45021 | 376.07 | 0.9752 |
| B4_2 | | | | 54466 | 376.16 | 0.9721 |
| B4_3 | | | | 71557 | 375.94 | 0.9730 |
| B5_1 | | MFCB | PSB+NFB[4] | 58807 | 376.01 | 0.9728 |
| B5_2 | | | | 54509 | 375.86 | 0.9740 |
| B5_3 | | | | 54591 | 375.88 | 0.9734 |
| B6_1 | | CK | CK | 53211 | 376.09 | 0.9737 |
| B6_2 | | | | 55118 | 375.68 | 0.9731 |
| B6_3 | | | | 73585 | 375.75 | 0.9728 |
| C1_1 | I_30 | M | PSB | 60018 | 376.04 | 0.9722 |
| C1_2 | | | | 63039 | 375.85 | 0.9694 |
| C1_3 | | | | 66388 | 376.00 | 0.9725 |
| C2_1 | | C | NFB | 64197 | 376.05 | 0.9734 |
| C2_2 | | | | 57061 | 375.98 | 0.9715 |
| C2_3 | | | | 64206 | 376.10 | 0.9744 |
| C3_1 | | MF | PSB | 48211 | 376.02 | 0.9675 |
| C3_2 | | | | 47685 | 375.79 | 0.9633 |
| C3_3 | | | | 48980 | 375.97 | 0.9627 |
| C4_1 | | CB | NFB | 53151 | 376.13 | 0.9650 |
| C4_2 | | | | 45751 | 376.02 | 0.9656 |
| C4_3 | | | | 54885 | 375.96 | 0.9635 |
| C5_1 | | MFCB | PSB+NFB | 44651 | 375.70 | 0.9678 |
| C5_2 | | | | 48346 | 376.02 | 0.9672 |
| C5_3 | | | | 52503 | 376.12 | 0.9623 |
| C6_1 | | CK | CK | 48299 | 375.95 | 0.9635 |
| C6_2 | | | | 46895 | 376.19 | 0.9680 |
| C6_3 | | | | 58524 | 376.48 | 0.9814 |

| | | | | | | |
|---|---|---|---|---|---|---|
| D1_1 | I_45 | M | PSB | 39946 | 376.35 | 0.9711 |
| D1_2 | | | | 47086 | 376.02 | 0.9644 |
| D1_3 | | | | 48179 | 376.20 | 0.9642 |
| D2_1 | | C | NFB | 57055 | 376.20 | 0.9662 |
| D2_2 | | | | 52573 | 376.10 | 0.9646 |
| D2_3 | | | | 46776 | 376.17 | 0.9622 |
| D3_1 | | MF | PSB | 55436 | 376.17 | 0.9668 |
| D3_2 | | | | 73796 | 376.11 | 0.9743 |
| D3_3 | | | | 60691 | 376.20 | 0.9684 |
| D4_1 | | CB | NFB | 62610 | 376.09 | 0.9714 |
| D4_2 | | | | 62047 | 376.20 | 0.9697 |
| D4_3 | | | | 73616 | 376.18 | 0.9748 |
| D5_1 | | MFCB | PSB+NFB | 45888 | 376.15 | 0.9608 |
| D5_2 | | | | 54539 | 376.16 | 0.9662 |
| D5_3 | | | | 53492 | 376.05 | 0.9635 |
| D6_1 | | CK | CK | 53381 | 376.09 | 0.9632 |
| D6_2 | | | | 56591 | 376.11 | 0.9659 |
| D6_3 | | | | 46532 | 376.09 | 0.9600 |
| F1_1 | II_45 | M | PSB | 59922 | 376.34 | 0.9664 |
| F1_2 | | | | 50301 | 376.26 | 0.9626 |
| F1_3 | | | | 49140 | 376.23 | 0.9577 |
| F2_1 | | C | NFB | 57647 | 376.31 | 0.9633 |
| F2_2 | | | | 49042 | 376.26 | 0.9610 |
| F2_3 | | | | 59258 | 376.29 | 0.9716 |
| F3_1 | | MF | PSB | 52737 | 376.27 | 0.9633 |
| F3_2 | | | | 55177 | 376.16 | 0.9657 |
| F3_3 | | | | 58206 | 376.25 | 0.9660 |
| F4_1 | | CB | NFB | 51058 | 376.47 | 0.9637 |
| F4_2 | | | | 49320 | 376.29 | 0.9662 |
| F4_3 | | | | 55687 | 376.19 | 0.9638 |
| F5_1 | | MFCB | PSB+NFB | 54793 | 376.34 | 0.9608 |
| F5_2 | | | | 43272 | 376.21 | 0.9532 |
| F5_3 | | | | 50481 | 376.16 | 0.9621 |
| F6_1 | | CK | CK | 38403 | 376.23 | 0.9469 |
| F6_2 | | | | 44836 | 376.29 | 0.9604 |
| F6_3 | | | | 44737 | 376.15 | 0.9580 |
| G1_1 | III_45 | M | PSB | 45469 | 376.35 | 0.9580 |
| G1_2 | | | | 53225 | 376.34 | 0.9625 |
| G1_3 | | | | 58104 | 376.26 | 0.9645 |
| G2_1 | | C | NFB | 58139 | 376.37 | 0.9666 |
| G2_2 | | | | 40303 | 376.48 | 0.9469 |
| G2_3 | | | | 52116 | 376.40 | 0.9608 |
| G3_1 | | MF | PSB | 58100 | 376.46 | 0.9655 |

| | | | | | | |
|---|---|---|---|---|---|---|
| G3_2 | | | | 62086 | 376.29 | 0.9681 |
| G3_3 | | | | 61197 | 376.44 | 0.9675 |
| G4_1 | | | | 64088 | 376.44 | 0.9670 |
| G4_2 | | CB | NFB | 36281 | 376.41 | 0.9509 |
| G4_3 | | | | 45265 | 376.32 | 0.9556 |
| G5_1 | | | | 47905 | 376.34 | 0.9641 |
| G5_2 | | MFCB | PSB+NFB | 55625 | 376.34 | 0.9643 |
| G5_3 | | | | 51043 | 376.38 | 0.9644 |
| G6_1 | | | | 68323 | 376.34 | 0.9697 |
| G6_2 | | CK | CK | 54653 | 376.38 | 0.9670 |
| G6_3 | | | | 51328 | 376.24 | 0.9622 |
| E1_1 | | | | 55370 | 376.33 | 0.9641 |
| E1_2 | | M | PSB | 49742 | 376.28 | 0.9597 |
| E1_3 | | | | 52131 | 376.34 | 0.9623 |
| E2_1 | | | | 62898 | 376.35 | 0.9706 |
| E2_2 | | C | NFB | 67019 | 376.42 | 0.9715 |
| E2_3 | | | | 49719 | 376.32 | 0.9615 |
| E3_1 | | | | 50326 | 376.37 | 0.9631 |
| E3_2 | | MF | PSB | 69329 | 376.35 | 0.9706 |
| E3_3 | | | | 51928 | 376.33 | 0.9609 |
| E4_1 | IV_45 | | | 55319 | 376.39 | 0.9646 |
| E4_2 | | CB | NFB | 45696 | 376.39 | 0.9587 |
| E4_3 | | | | 48518 | 376.43 | 0.9600 |
| E5_1 | | | | 48462 | 376.41 | 0.9613 |
| E5_2 | | MFCB | PSB+NFB | 52052 | 376.35 | 0.9632 |
| E5_3 | | | | 51562 | 376.45 | 0.9620 |
| E6_1 | | | | 47230 | 376.43 | 0.9580 |
| E6_2 | | CK | CK | 51153 | 376.46 | 0.9593 |
| E6_3 | | | | 50983 | 376.31 | 0.9615 |

After quality filtering, 112 samples yielded a total of 5,985,527 16S rRNA gene sequences with an average of 53,442 reads per sample. The length of the trimmed sequences ranged between 360 bp and 400 bp. The treatments are: 0d: soil sampled before bio-fertilization; M or C: single application of *Bacillus megaterium* or *Azotobacter chroococcum*; MF: dual application with *B. megaterium* and *Pseudomonas fluorescens*; CB: dual application with *A. chroococcum* and *Azospirillum brasilence*; MFCB: application with four strains; CK: non-inoculation. The sampling day are: I-10, I-30 and I-45: 10 days, 30 days, and 45 days after the first bio-fertilization, respectively. II-, III-, and IV-45: 45 days after the second, third, and fourth bio-fertilization, respectively.

[1] Coverage: Good’s non-parametric coverage estimator.

[2] PSB: phosphate solubilizing bacteria

[3] NFB: nitrogen fixing bacteria

[4] PSB+NFB: combined with PSB and NFB

**Table S2** Effects of sampling time and treatments on the OTUs, diversity and richness.

| Treatments | Sampling time | | | | | |
|---|---|---|---|---|---|---|
| | **I-10** | **I-30** | **I-45** | **II-45** | **III-45** | **IV-45** |
| | | | *OTUs* | | | |
| | B | A | A | A | A | A |
| **M** | 2874 | 3673.3 | 3279.7ab | 3097.7 | 2953 | 3190.7 |
| **MF** | 3027.7 | 3304 | 3643.7a | 3050.7 | 3113 | 3220 |
| **C** | 2925 | 3807 | 3226ab | 3326.3 | 2933.3 | 3355.7 |
| **CB** | 3016 | 3221.7 | 3257.3b | 3142.7 | 2948.3 | 3133.3 |
| **MFCB** | 3100.7 | 3190 | 3171ab | 3086.7 | 2916 | 3155.7 |
| **CK** | 3275.3 | 3472.7 | 3296.7ab | 3055.7 | 3209.7 | 3209.7 |
| | | | *Shannon* | | | |
| **M** | 6.02B | 6.69bA | 6.65abA | 6.58A | 6.72A | 6.78A |
| **MF** | 6.25B | 6.69abAB | 6.76abA | 6.6AB | 6.72AB | 6.77A |
| **C** | 6.04B | 6.79aA | 6.74aA | 6.77A | 6.72A | 6.76A |
| **CB** | 6.07B | 6.58abA | 6.51bAB | 6.62A | 6.79A | 6.76A |
| **MFCB** | 6.13B | 6.64abA | 6.63bA | 6.62A | 6.73A | 6.79A |
| **CK** | 6.42B | 6.77abAB | 6.7aAB | 6.73AB | 6.75AB | 6.84A |
| | | | *Simpson* | | | |
| **M** | 0.0225abA | 0.004B | 0.0045B | 0.0078AB | 0.0033B | 0.0029B |
| **MF** | 0.0088a | 0.0035 | 0.0042 | 0.0063 | 0.0032 | 0.0029 |
| **C** | 0.0133ab | 0.0033 | 0.0031 | 0.0045 | 0.0034 | 0.0031 |
| **CB** | 0.0159a | 0.0052 | 0.0049 | 0.0055 | 0.0027 | 0.003 |
| **MFCB** | 0.0123a | 0.0044 | 0.0047 | 0.0092 | 0.0031 | 0.0028 |
| **CK** | 0.0057b | 0.0031 | 0.0038 | 0.0057 | 0.0036 | 0.0027 |
| | | | *Ace* | | | |
| **M** | 4406.3b | 5069.1ab | 4596.6 | 4345.9 | 4156.9b | 4539.7 |
| **MF** | 4339.7b | 5274.3ab | 5037.6 | 4271.3 | 4327.4a | 4511.9 |
| **C** | 4674.9a | 5215.6a | 5098.4 | 4512.1 | 4193.6ab | 4645.3 |
| **CB** | 4290.2b | 4621.2ab | 4892.5 | 4394.5 | 4184.5ab | 4678.3 |
| **MFCB** | 4663.8ab | 4514.1b | 5027.8 | 4376.9 | 4023.5b | 4415.9 |
| **CK** | 5186.2a | 4723.4ab | 4663.4 | 4343.6 | 4420.7ab | 4549.6 |
| | | | *Chao1* | | | |
| **M** | 4151.6b | 5118a | 4662.5ab | 4340.8 | 4129a | 4534.5 |
| **MF** | 4398.9aB | 4822.1bAB | 5072.4aA | 4224.5AB | 4246.8aAB | 4620.5AB |
| **C** | 4259.3abB | 5162.9aA | 4698.8abAB | 4451AB | 4135.9abAB | 4623.3A |
| **CB** | 4280.0abB | 4628.3bA | 4668.6abAB | 4368AB | 4090.3abAB | 4452.7A |
| **MFCB** | 4508.9a | 4545.2ab | 4645.4b | 4317.3 | 3928.1b | 4432 |
| **CK** | 4814.6a | 4693.9ab | 4662.5b | 4301.6 | 4348.7a | 4570 |

Values within the same column followed by the different lowercases indicate significant difference ($P < 0.05$) among treatments, capital letters indicate significant difference ($P < 0.05$) among sampling time by Tukey's test. The treatments were M or C: single application of *Bacillus megaterium* or *Azotobacter chroococcum*, respectively; MF: dual application of *B. megaterium* and *Pseudomonas fluorescens*; CB: dual application of *A. chroococcum* and *Azospirillum brasilense*; MFCB: application of all four strains; CK: non-inoculated treatment.

**Table S3** Overall ANOVA test results for the whole treatments and sampling time

| ANOVA test | Shannon | Simpson | Ace | Chao |
|---|---|---|---|---|
| Treatment | ns | ns | ns | * |
| Day | **** | **** | ** | **** |
| Treatment × day | ns | ns | ns | ns |

"ns" means no significance. Significant difference: *p≤0.05; **p≤0.01; ***p≤0.001. **** p≤ 0.0001.

**Table S4** Significance between all sampling time for each treatment and significance between all treatments for each sampling time by Kruskal-Wallis H test.

| Phyla | Significance between six sampling time for each treatment | | | | | |
|---|---|---|---|---|---|---|
| | **M** | **MF** | **C** | **CB** | **MFCB** | **CK** |
| *Proteobacteria* | | ** | * | | ** | |
| *Acidobacteria* | ** | *** | *** | *** | *** | *** |
| *Bacteroidetes* | * | ** | *** | ** | ** | ** |
| *Actinobacteria* | | * | | | | |
| *Chloroflexi* | ** | *** | ** | *** | ** | ** |
| *Gemmatimonadetes* | * | ** | ** | ** | ** | * |
| *Planctomycetes* | * | * | *** | ** | | ** |
| *Cyanobacteria* | | | * | * | ** | * |
| *Firmicutes* | * | | * | | | |
| *Verrucomicrobia* | *** | * | | | * | ** |
| *Armatimonadetes* | * | *** | ** | *** | ** | ** |
| **Phyla** | **Significance between six treatments for each sampling time** | | | | | |
| | **I-10** | **I-30** | **I-45** | **II-45** | **III-45** | **IV-45** |
| *Proteobacteria* | | | | | | |
| *Acidobacteria* | | $C^+$ | | $C^+$ | | |
| *Bacteroidetes* | | | | | | |
| *Actinobacteria* | | | | | | |
| *Chloroflexi* | | | | | | |
| *Gemmatimonadetes* | | | | | | |
| *Planctomycetes* | | | | | $M^-$, $MF^-$ | $M^-$, $C^-$ |
| *Cyanobacteria* | | | | $CK^-$ | | |
| *Firmicutes* | | | | | | |
| *Verrucomicrobia* | | | | | | |
| *Armatimonadetes* | | | | | | $MF^+$ |

"ns" means no significance. Significant difference: *$p \leq 0.05$; **$p \leq 0.01$; ***$p \leq 0.001$.

$C^+$ : The abundance of *Acidobacteria* was increased in treatment C but decreased in treatments by comparing to CK

$M^-$, $MF^-$, $C^-$ :The abundance of *Planctomycetes* was decreased in treatment M, MF and C by comparing to CK

$CK^-$ : The abundance of *Cyanobacteria* was decreased in CK by comparing to other treatments

$MF^+$: The abundance of *Armatimonadetes* was decreased in treatment MF by comparing to CK;

The treatments are: M or C: single application of *Bacillus megaterium* or *Azotobacter chroococcum*; MF: dual application with *B. megaterium* and *Pseudomonas fluorescens*; CB: dual application with *A. chroococcum* and *Azospirillum brasilence*; MFCB: application with four strains; CK: non-inoculation. The sampling day are: I-10, I-30 and I-45: 10 days, 30 days, and 45 days after the first bio-fertilization, respectively. II-, III-, and IV-45: 45 days after the second, third, and fourth bio-fertilization, respectively.

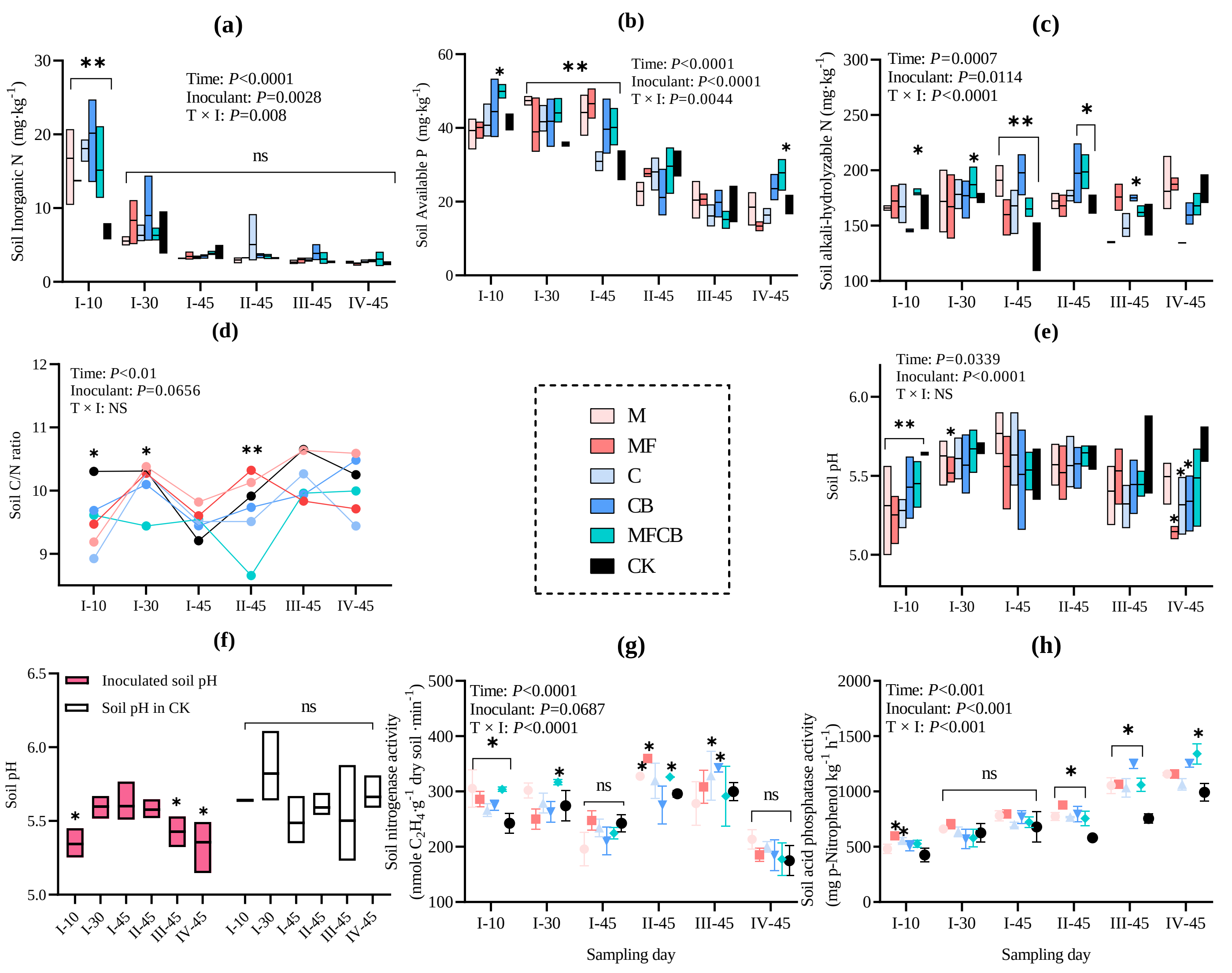


**Fig. S1** Responses of (**a**) soil inorganic N, (**b**) soil available P, (**c**) soil alkali-hydrolyzable N, (**d**) C/N ratio, (**e**) soil pH, (**f**) average soil pH under inoculation and control, (**g**) soil nitrogenase activity, and (**h**) soil acid phosphate activity to inoculant types and sampling time. “ns” means no significance. Significant difference: *p≤0.05; ** p≤ 0.01. The treatments are: M or C: single application of *Bacillus megaterium* or *Azotobacter chroococcum*; MF: dual application with *B. megaterium* and *Pseudomonas fluorescens*; CB: dual application with *A. chroococcum* and *Azospirillum brasilence*; MFCB: application with four strains; CK: non-inoculation. The sampling day are: I-10, I-30 and I-45: 10 days, 30 days, and 45 days after the first bio-fertilization, respectively. II-, III-, and IV-45: 45 days after the second, third, and fourth bio-fertilization, respectively.

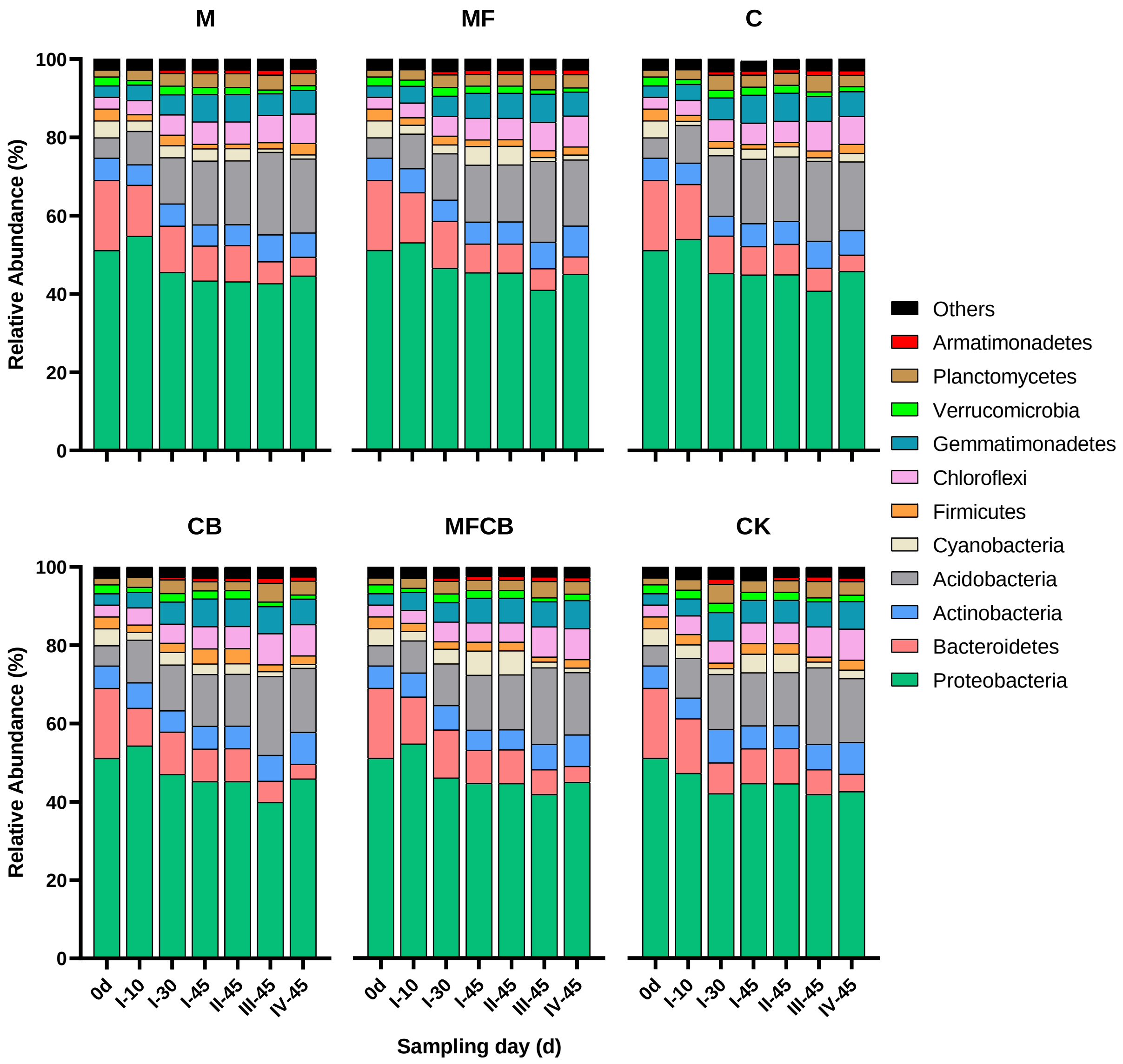


**Fig. S2** Relative abundance (%) of soil microbial phyla in soils under different treatments. The value of each bacterial group percentage is the mean of soil samples collected from three different replicates. The treatments are: M or C: single application of *Bacillus megaterium* or *Azotobacter chroococcum*; MF: dual application with *B. megaterium* and *Pseudomonas fluorescens*; CB: dual application with *A. chroococcum* and *Azospirillum brasilence*; MFCB: application with four strains; CK: non-inoculation. The sampling day are: 0d: the soil samples before the bio-fertilization; I-10, I-30 and I-45: 10 days, 30 days, and 45 days after the first bio-fertilization, respectively. II-, III-, and IV-45: 45 days after the second, third, and fourth bio-fertilization, respectively

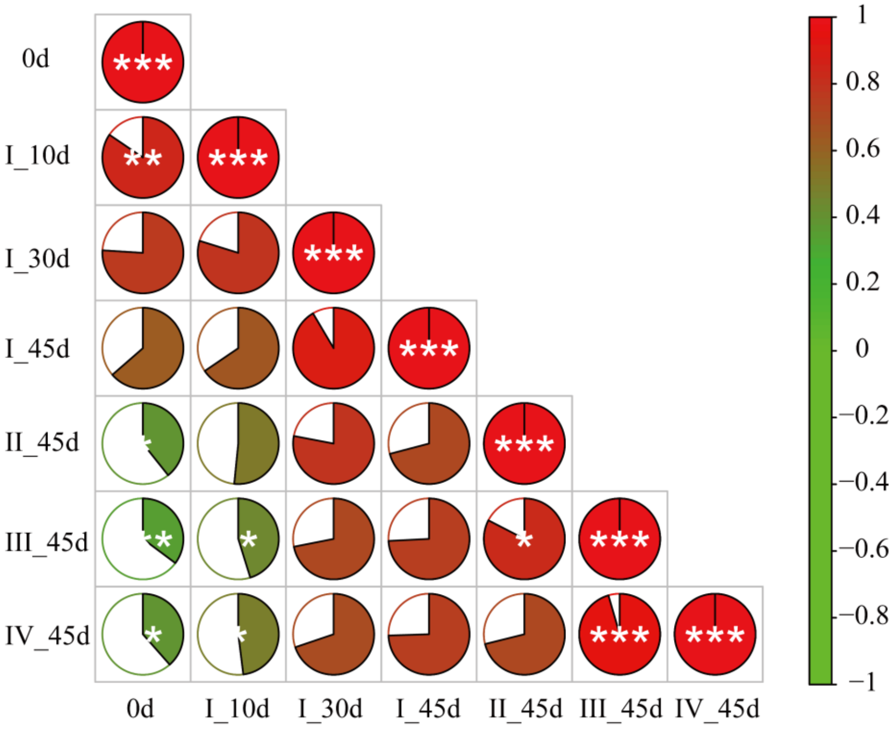


**Fig. S3** Pairwise correlations of whole soil microbiome between time points. The sampling day are: 0d: the soil samples before the bio-fertilization; I-10, I-30 and I-45: 10 days, 30 days, and 45 days after the first bio-fertilization, respectively. II-, III-, and IV-45: 45 days after the second, third, and fourth bio-fertilization, respectively. Significant difference: *p≤0.05; ** p≤ 0.01, *** p≤ 0.001.

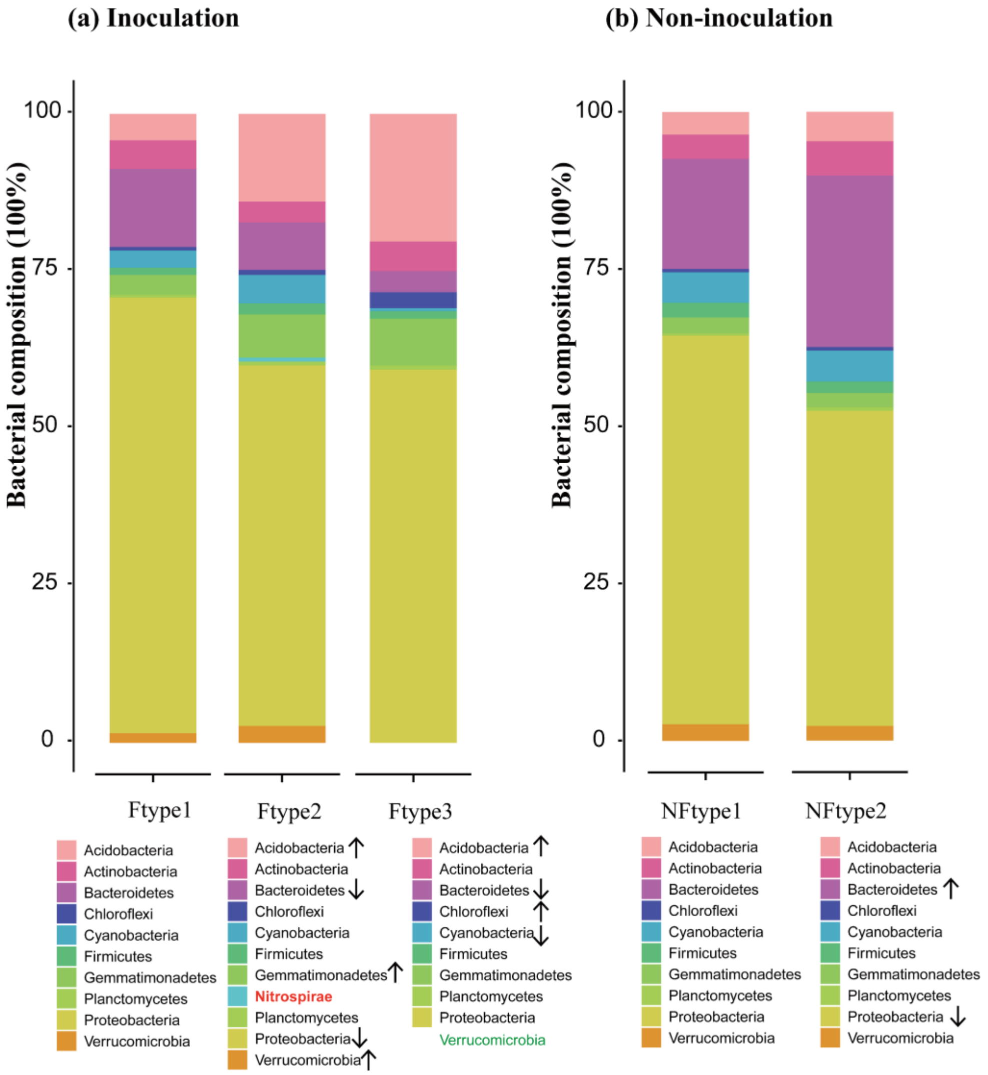


**Fig. S4** Community composition of different cluster types in (a) inoculated and (b) non-inoculated soils. " ↑ " means the phylum significantly increased compared to that in the former cluster type, " ↓ "means the phylum significantly decreased compared to that in the former cluster type. The phylum name in red means a new phylum was detected compared to the first type; the phylum name in green means the phylum disappeared compared to the first type.

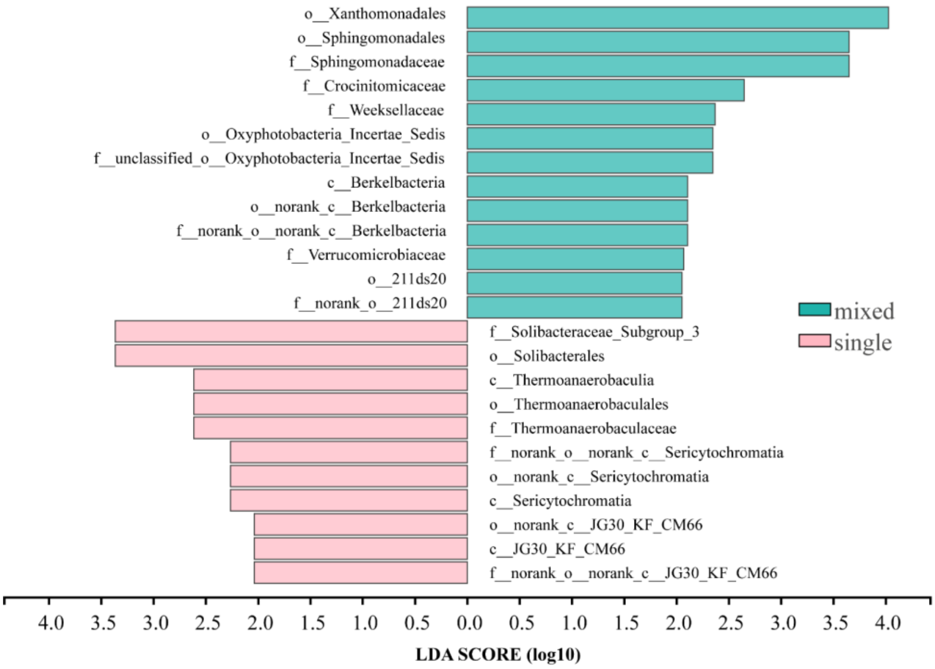


**Fig. S5** Linear discriminant analysis of abundances from order to family level between single inoculant (M, C) and mixed inoculants (MF, CB, MFCB) at 30 days after the first PGPR inoculation.
